# The Health Profile of Populations Living in Contaminated Sites: Sentieri Approach

**DOI:** 10.1155/2013/939267

**Published:** 2013-06-18

**Authors:** Roberta Pirastu, Roberto Pasetto, Amerigo Zona, Carla Ancona, Ivano Iavarone, Marco Martuzzi, Pietro Comba

**Affiliations:** ^1^Department of Biology and Biotechnologies Charles Darwin, Sapienza University of Rome, Piazzale Aldo Moro, 5-00185 Rome, Italy; ^2^Department of Environment and Primary Prevention, National Health Institute, Viale Regina Elena, 299-00161 Rome, Italy; ^3^Department of Epidemiology, Lazio Regional Health Service, Via di Santa Costanza, 53-00198 Rome, Italy; ^4^WHO European Centre for Environment and Health, Bonn Office, Hermann-Ehlers-Straße 10, 53113 Bonn, Germany

## Abstract

SENTIERI project (Epidemiological Study of Residents in Italian Contaminated Sites) studied mortality in the sites of national interest for environmental remediation (National Priority Contaminated Sites—NPCSs). SENTIERI described mortality of residents in NPCSSs, and it specifically focused on causes of death for which environmental exposure is suspected or ascertained to play an etiologic role. The epidemiological evidence of the causal association was classified *a priori* into one of these three categories: Sufficient (S), Limited (L), and Inadequate (I). Mortality in the period 1995−2002 was studied for 63 single or grouped causes at the municipal level by computing: crude rate, standardized rate, standardized mortality ratios (SMR), and SMR adjusted for an *ad hoc* deprivation index. Regional populations were used as references for SMR calculations and 90% CI accompanied SMR values. The deprivation index was constructed using 2001 national census variables for the following socioeconomic domains: education, unemployment, dwelling ownership, and overcrowding. SENTIERI results will allow the priorities setting in remediation intervention so as to prevent adverse health effects from environmental exposure. This paper's objective is to present the rationale, methods, advantages, and limitations underlying SENTIERI project and to describe data and resources required to apply a similar approach in other countries.

## 1. Introduction

Human health is intimately connected to the surrounding environment. This is particularly the case of the health of people living in contaminated site(s) (CS) which is affected by the legacy of past industrialization and current industrial activities, often in absence of environmental remediation.

European Community legislation addresses the concept of CS only in the context of the Thematic Strategy for Soil Protection [1] and the Soil Framework Directive proposed by the European Commission (EC) in 2006 [2]; for details on the legal framework and definitions at EU level refer to WHO 2013 [3].

According to 2007 estimates, soil contamination requiring clean up is present in approximately 250,000 sites in the European Environment Agency (EEA) member countries [4]. Although main polluting sources may vary across Europe, industrial production and commercial activities, oil industry and waste disposal and treatment are reported to be the major ones. National reports indicate that heavy metals and mineral oils are the main soil contaminants, while mineral oils and chlorinated hydrocarbons are the most frequent pollutants found in groundwater [5].

The term CS can have different meanings. A general definition, following the public health perspective, is “areas hosting or having hosted human activities which have produced or might produce environmental contamination of soil, surface or groundwater, air, and food chain, resulting or being able to result in human health impacts” [3]. Given this definition, an area affected by a single chemical contamination of a single environmental matrix (e.g., the soil contamination caused by a given pesticide) and a large area with soil, water, air, and food chain contamination by multiple chemicals (e.g., the contamination caused by long-term emissions of a petrochemical complex) can be both considered contaminated sites. 

There are several approaches and methods for assessing the health impact of National Priority Contaminated Sites (NPCS). On the one hand, one can apply risk assessment techniques, where available data on the degree of contamination (typically measures of concentration of specific hazardous chemical agents) are used to quantitatively estimate the risk of occurrence of health endpoints causally associated with the agents; on the other hand, epidemiological approaches applicable to NPCSs involve the inclusive collection of available health information of resident population, and various agents and exposures. In these assessments, a first descriptive level is based on epidemiological tools that do not require an *ad hoc* collection of data and aims at describing the health profile of populations documenting ascertained or suspected associations with local environmental risks and the potentiality to provide efficient answers. More detailed analyses can be carried out, at a higher level of definition, by collecting specific data on health outcomes and/or on exposure [3]. 

SENTIERI project (Epidemiological Study of Residents in Italian Contaminated Sites) [6, 7] is an example of first level descriptive approach adopting an ecological study design, looking at the aggregate population level rather than at individual level. SENTIERI project is a national project developed to evaluate the health profile of populations residing in the Italian sites of national interest for environmental remediation-National Priority Contaminated Sites (NPCS). These sites were labelled as sites of national interest because of their substantial contamination, documented in qualitative and/or quantitative terms, and the consequent potential impact on the health of residents.

The methods proposed under the approach exemplified by SENTIERI can be generalized and applied to other NPCSs. 

This paper's objective is to present the rationale and methods underlying SENTIERI project and to describe data and resources required to apply a similar approach in other countries. 

## 2. Rationale

When studying how the environment can adversely affect human health, it is usually very difficult to identify clear cause-effect relationships because they are characterized by multicausality with different strengths of association. In addition, these relationships are influenced by individual factors (e.g., genetic, diet, life-style, occupation, and socioeconomic status) that can also have a role on both exposure level and disease development. SENTIERI project was developed to deal with this complex scenario. It describes the health profile of residents in contaminated sites through small area analysis by applying the multistep procedure described in the following sections. A selection of epidemiologic terms are defined in Glossary to facilitate the reading of the paper.

## 3. The Study of the Health Profile of Residents in NPCSs: What to Do and Why

### 3.1. Site Selection

As a first step, NPCSs to be studied should be chosen, and the criteria adopted to define NPCS/NPCSs are clearly indicated. The NPCS selection will depend on the aims of the study to be undertaken, on the availability of the NPCCs-related information, and on any other consideration researchers would make and consider appropriate. In many instances, NPCSs to be studied are chosen by third parties, such as an environmental authority, by public concern, and media pressure. It is advisable that the criteria used throughout this phase are clearly stated.

### 3.2. Environmental Data

In most instances NPCSs are characterized by the presence of numerous and different environmental sources of contamination possibly leading to human exposures. All the available NPCS data should be collected and described in a standardized, homogeneous way. Geographical characteristics, extension of the contaminated area, and demographic information about residents potentially affected should be listed. Detailed description of contamination characteristics should be included as well as the presence of industries and all other human activities that have contributed to the environmental deterioration of the NPCS. Researchers will specify the sources used for this task: scientific reports, acts, and so on.

### 3.3. Study Population

Criteria to define populations affected by contamination may vary. Populations at risk can be identified as people living in the neighborhood of NPCSs sources or living in areas defined as contaminated. Typically, the distance from the areas affected by contamination is used, but also dispersion modelling results are used. In the latter case, the definition of the areas most affected by contamination, and the consequent identification of at-risk populations, depends on the accuracy of the model. There are several models used to evaluate the areas affected by contaminants; their implementation and improvement depend on the available information on several parameters. For example, in the case of emissions into the air from oil refineries, the parameters to be considered should be characteristics of emission sources: for example, height, flow rate, composition of emissions, exit temperature, local orography, and meteorological conditions [8]. Areas characterised by contamination processes different from direct industrial emissions require other parameters. In the case of landfills many early disposal sites did not have liners to trap rainwater that percolate through the landfill, and some newer landfills have liners that leak. The percolating water leaches toxic chemicals (e.g., from batteries, electronic equipment, and discarded household chemicals) that can contaminate soil and groundwater in ways that make it difficult to adequately identify the population at risk. Furthermore, in defining contaminated areas in case of complex industrial contamination, it should be considered that populations can experience several routes of exposure, mainly through inhalation of pollutants emitted into the atmosphere, and through ingestion when contaminants are accumulated in soil, water, and food chain. 

### 3.4. Reference Population

For the reference population the same data of the area units under study are needed: cases and populations stratified by gender and age categories.

The reference population should be selected considering two different needs: (1) it should be comparable to the studied populations for factors that can affect the health profile with the exception of the contamination at study—the differences in the health profile between the compared populations should be ideally due only to the differences in environmental exposures, namely, to the contamination; (2) it should be sufficiently numerous to obtain stable reference rates also for rare diseases. These two needs have opposite requirements, as the first one is usually negatively correlated with the dimension of the population, while the second one is positively correlated with the dimension of the population. The reference populations should be selected balancing these two needs. Usually one or two populations among the following are selected as reference population: national, and regional, local (i.e., a population composed of populations residing in the neighbourhood of the contaminated area).

### 3.5. Outcome Selection

The aims of the study will imply a sound outcomes selection to include the ones for which environmental exposure/s (see environmental data) is suspected or ascertained to play an etiologic role. The possible health impact from environmental exposures is measured in terms of mortality, morbidity, incidence of neoplastic diseases, and so forth. General considerations about the quality of available information and data as well as intrinsic limitations of the selected outcome measures should be described and discussed.

Health indicator characteristics should be carefully examined and multiple aspects considered taking into account the inherent uncertainty. Sources of national or local routinely collected data, spatial and temporal coverage, and quality aspects are all of extreme importance for the validity of study results and their usefulness in terms of general knowledge and public health relevance.

An appropriate length of the period under study will make research results and conclusions more informative for diseases with long latency times; precision of the epidemiological parameters will also improve with a longer study period. 

### 3.6. Small-Area Studies

Small-area studies investigate the role of neighbourhood level [9]. The specific value of small-area analysis is that it permits the examination of data for population which tend to be more homogeneous in character and in their environmental circumstances than the larger and more widely spread populations [10]. 

The smallest territorial unit that can be used in small area studies depends on data availability that may vary in different countries. For example, in Italy small area studies can be carried out at the census tract (average of 200 residents) and municipality levels. In 2001 about 70% of the Italian municipalities included less than 5,000 residents; this compares with Great Britain, where small area studies can be carried out at the following levels: enumeration districts—400 residents, electoral wards—5,100 inhabitants, and postcode sectors—6,600 people [11].

### 3.7. Socioeconomic Confounding

In geographical studies of environment and health, confounding from social and economic factors may occur. To control such confounding effect, standardisation techniques have been extensively used since the mid-1990s. To account for possible confounding from socioeconomic factors in SENTIERI project, an *ad hoc *Deprivation Index was built and applied to the SMR estimates.

Deprivation can be defined as “a state of observable and demonstrable disadvantage relative to the local community or the wider society or nation to which an individual, family, or groups belong” [12]. Deprivation indices are area-based measures of material and social disadvantageous circumstances, that is, indicators of relative deprivation at population level. A detailed discussion on the use and critical aspects of the application of DI in small area studies of environment and health can be found in an open access systematic review [11]. 

### 3.8. *A Priori* Evaluation of the Evidence

When performing epidemiologic studies, there is a risk for researchers to become data-driven. This can be the case when commenting results for causes showing an increase, possibly on the basis of statistical significance. As an example, SENTIERI dealt with the complexity of the relation between area contamination and health effects. For each NPCS studied the project focused on those causes identified *a priori* from the epidemiological evidence of their association with selected environmental exposures. This is the basic key aspect of the SENTIERI approach.

It is suggested that researchers use environmental contaminants information considering the available level of details. In SENTIERI possible relevant exposures were abstracted from Legislative Decrees, that is, administrative sources defining NPCSs boundaries and coded on a productive sectors basis (i.e., petrochemicals and/or refineries, harbours areas, etc.). The choice was made because NPCSs had heterogeneous level of environmental characterization. While for some NPCSs information on specific chemical contaminants was available, for others only productive plants were listed. This is to point out that researchers should be able to adapt this approach to their specific situation.

When studying factors that determine changes in the occurrence of a given health condition or its predictive factors, it should always be kept in mind that most diseases have a multiple etiology. Therefore also exposures different from those specifically present in NPCSs should be considered, as well as every known cause or risk factor, such as smoking habits, alcohol consumption or other sources independent from those at study, that is, socioeconomic factors and occupational exposures, and air pollution due to combustion of fuels for transport activities and domestic heating. The latter aspects are particularly relevant when studying complex industrial settings close or overlapping to urban highly populated areas.

Once the environmental exposures of interest identified, researchers should examine the updated scientific literature to evaluate the associated health effects. This apparently easy task is in fact quite demanding, because by browsing the literature different kinds of publications are collected: handbooks, original articles, letters to scientific journals, multicentric studies, editorials, reviews, meta-analyses, and so on. 

Therefore, the first decision to be taken is about the “relevance” to give to the collected material and how to use it to define the strength of the association between the specific health outcome and the environmental exposure/s that characterize/s the NPCSs. 

SENTIERI study group defined a hierarchy in the literature sources. Sources expressing the epidemiological community consensus, evaluating scientific evidence by means of standardized criteria, and weighting the study design and the occurrence of biased results were considered most important (i.e., IARC monographs, WHO publications, European Environment Agency reports, handbooks of environmental, and occupational medicine). They were followed in the hierarchy by quantitative meta-analyses. Multi-centric studies, systematic reviews, and single investigations were also considered. Consistency among sources was a criterion used to classify the strength of the causal association. 

This process was performed considering not only the environmental exposures, but also those risk factors previously mentioned (smoking habits, alcohol consumption, air pollution, socioeconomic aspects, and occupational exposures). Literature sources were presented in the final report in a tabular form to let the reader follow the entire process of evaluation for each cause combined with different exposures. On the basis of explicit criteria the strength of the causal association for each cause-exposure combination was classified (matrix of *a priori* evidence evaluation).

The complexity of the described approach requires a multidisciplinary group of researchers. Expertise in environmental and occupational epidemiology, statistics, and public health clinical medicine, toxicology, and analytical chemistry are needed to tackle this complex environmental issue.

## 4. The Study of the Health Profile of Residents in NPCSs: SENTIERI Project

### 4.1. Site Selection

As indicated previously, the location of NPCSs should be identified. All data about contamination sources and characterization of contamination should be collected and examined to identify the contamination diffusion and finally define the areas and populations possibly affected by contaminants. SENTIERI project studied populations residing in the sites of national interest for environmental remediation (National Priority Contaminated Sites—NPCSs). 

### 4.2. Environmental Data

In SENTIERI the production activities and sources of contamination listed in the Decrees defining the sites' boundaries were used as a proxy for environmental residential exposures; they are coded as chemical industries, petrochemicals and refineries, steel plants, power plants, mines and/or quarries, harbour areas, and asbestos or other mineral fibres, landfills, and incinerators. For each NPCS contamination data were collected, both from the national and local environmental remediation programmes.

### 4.3. Study Population

The study population comprised residents in 44 NPCSs; each one included one or more municipalities, a total of 5.5 million inhabitants in the 44 NPCSs, about 10% of the Italian population at the time of 2001 Census.

### 4.4. Reference Population

In SENTIERI project the Italian 2001 standard population was used as a reference to calculate crude and standardized rates. Regional populations were used as reference in the indirect standardization.

### 4.5. Outcome Selection

Mortality for 63 causes (or group of causes) for the period 1995–2002.

SENTIERI approach can be applied considering different health outcomes. Each outcome and cause analysed should be characterized to define its specific contribution in evaluating the possible health impact of contamination. For this aim, mortality, morbidity (e.g., hospital discharge records), cancer incidence, and congenital malformations prevalence could be of major interest. As described in the previous section, for each outcome a matrix of *a priori *evaluation should be built. Each outcome can be considered usable for the SENTIERI approach if its validity is previously verified, and appropriate epidemiological parameters can be estimated.

### 4.6. Small-Area Statistics

Briefly, SENTIERI studied mortality in 44 NPCSs using data at municipality level (1995–2002), calculating indicators such as crude and standardized rates (Italian 2001 standard population as reference) and standardized mortality ratio (SMR), using regional comparison rates, both crude (SMR) and adjusted for an *ad hoc* deprivation index (SMR ID).

### 4.7. Socioeconomic Confounding

To control for confounding from social and economic factors an *ad hoc* Deprivation Index was built and applied to the SMR estimates in SENTIERI project (SENTIERI DI). The deprivation index was constructed using the 2001 national census variables representing the following socioeconomic domains: education, unemployment, dwelling ownership, and overcrowding.

### 4.8. *A Priori* Evaluation of the Evidence

The epidemiological evidence was examined on the basis of explicit criteria to build a matrix of the *a priori* epidemiological evaluation of the strength of the causal association for each combination of selected outcome and environmental exposure. For details concerning the *a priori *evaluation see Section 3.

The procedure to develop such matrix can be summarized as follows.

 Firstly a multidisciplinary group of researchers (see the previous section) draws up the list of causes to be submitted to the evidence evaluation.
 Secondly, the epidemiologists classify the strength of the causal association of each outcome/exposure combination.

To complete this phase in SENTIERI project, the epidemiologists developed a procedure to examine the epidemiological literature published from 1998 to 2009 on the health risk of populations living in NPCSs. For details refer to Section 3 and [6]. 

The epidemiologists examined each cause of death/exposure combination (mortality was the first outcome to be analyzed) in terms of strength of causal inference. “Environmental exposures” were distinguished from “other exposures” in NPCSs. The former were fixed on the basis of the possible sources of contamination in NPCSs listed in the Decrees that defined each site boundaries (e.g., chemical industry, steel plants); the “other exposures” were the environmental, lifestyle and occupational most important known etiological factors: air pollution, active and passive smoking, alcohol intake, occupational exposures, and socioeconomic status. The procedure finally led to classify the evidence of the causal association into three categories: Sufficient to infer the presence of a causal association, Limited to infer the presence of a causal association, and Inadequate to infer the presence or the absence of a causal association.

The criteria adopted for the classification are reported in Table 1.

At the end of the second phase, a matrix of epidemiological *a priori* evidence about the strength of each outcome-cause/*environmental exposure* or outcome-cause/“other exposure” causal association was prepared (example in Table 2).

## 5. The Study of the Health Profile of Residents in NPCSs: SENTIERI Project—Main Findings and Results for a Single Site and for All NPCSs Combined

Specific causes with a certain level of strength of causal association with the environmental exposures present in each NPCS were reported and discussed in detail in SENTIERI. In order to have a general description of the residents' health profile, main broad groups of causes of death were also considered. 

The assessment and appropriate consideration of previous studies performed on the same NPCS, if any, ameliorate the level of knowledge, reducing scientific uncertainties about the heath impact of contamination and facilitating the process of identification and implementation of remediation interventions.

When more than one NPCS are studied, a homogeneous way of presenting and discussing results can make the study results clearer and more readable. 

SENTIERI study group prepared a form to present each NPCS at study. It included a summary description of population and contamination data: specific sections dedicated to (a) results by gender (general health profile and specific, *a priori* selected causes); (b) previous studies carried out in the investigated area; and (c) a conclusive paragraph with suggestions for further scientific and/or remediation priorities as well as recommendations for public health interventions. 

In single sites examples of recommendations aimed at a better description and clarification of the observed health effects include the investigation of the respiratory diseases prevalence in children, the conduction of occupational and residential cohort studies, and health surveillance activities as well as exposure assessment and biological monitoring investigations. 

An example is the one of Sassuolo-Scandiano NPCS presented in a following paragraph.

### 5.1. Main Findings of SENTIERI Project

Some NPCS-specific results are noteworthy. The presence of asbestos or asbestiform fibres was the motivation for including six NPCSs in the “national environmental remediation programme.” In five of these sites increases in malignant pleural neoplasm mortality were observed; in four of them the excess was in both genders. In four out of six other sites where in addition to asbestos other sources of environmental pollution were reported, mortality from malignant pleural neoplasm was increased in both genders. Asbestos and pleural neoplasm represents a unique case. Unlike mesothelioma, most causes of death analyzed in SENTIERI have multifactorial etiology; furthermore in most NPCSs multiple sources of different pollutants are present, sometimes concurrently with air pollution from urban areas: in these cases, drawing conclusions on the association between environmental exposures and specific health outcomes might be a hard task.

Notwithstanding these difficulties, in a number of cases an etiological role could be attributed to some environmental exposures. The attribution could be possible on the basis of the increases observed in both genders and in different age classes, and the exclusion of a major role of occupational exposures was thus allowed (in Italy most of the workforce in industrial setting is still represented by males). For example, a role of emissions from refineries and petrochemical plants was hypothesized for the observed increases in mortality from lung cancer and respiratory diseases in two NPCSs; a role of emissions from metal industries was suggested to explain increased mortality from respiratory diseases in two other sites. In six NPCSs an etiological role of air pollution in the raise of congenital anomalies and perinatal disorders was suggested, and a causal role of heavy metals, PAH's, and halogenated compounds was suspected for mortality from renal failure in six sites.

### 5.2. Sassuolo-Scandiano: An Example for a Single SENTIERI Site

According to the 2001 Census, the NPCS Sassuolo-Scandiano includes 6 municipalities with a 102,811 overall population.

The perimetration Decree of this NPCS lists the presence of pottery manufacturing plants, an environmental exposure that SENTIERI qualifies as “C” (plants producing chemicals and/or chemical products).

SENTIERI results among the main causes of death show in this NPCS some excesses for all causes and circulatory and respiratory systems diseases in men. Some excesses are also observed for digestive system diseases in women (Table 3). For the causes of death with an *a priori* Sufficient or Limited evidence of causal association with the environmental exposure in this NPCS, an excess for respiratory diseases and asthma was observed in men (Table 4), and for congenital anomalies (malformations) in all age classes, both genders (Table 5).

Previous studies in this NPCS showed that lead, a metal used in the pottery production, polluted subsoil, surface, and ground waters. Notwithstanding a decrease in occupational exposure between the beginning of the Seventies and half of the Nineties, in 1995, the lead levels remained high. At the beginning of the Eighties, lead exposure levels in children were high; during the second half of the Nineties a sharp decrease was observed, and blood concentrations were lower than the 10 *μ*g/100 mL limit. According to SENTIERI, the evidence of the causal association between occupation and respiratory system diseases and asthma was classified as Sufficient. 

Since silicosis was not separately analyzed in SENTIERI and its risk is known to increase in pottery production, as observed in the cohort study of workers compensated for silicosis in Italy, it was probably conducive to the observed respiratory diseases excess in this NPCS. 

The occupational exposure to lead in pottery production might have contributed to the mortality excess due to Parkinson's disease and hypertension. SENTIERI observed an excess in men (SMR = 160 (90% C.I. 111–224), SMR ID = 168 (90% C.I. 117–234)) and similarly in women (SMR = 110 (90% C.I. 69–167), SMR ID = 110 (90% C.I. 69–166)). A hypertension excess was pointed out for both genders (men: SMR = 192 (90% C.I. 166–221), SMR ID = 190 (90% C.I. 164–219); women: SMR = 206 (90% C.I. 184–230), SMR ID = 196 (90% C.I. 176–219)).

In conclusion, data acquisition for the appraisal of the present lead environmental pollution and occupational exposure is suggested. Individual epidemiological investigations are recommended to study asthma prevalence.

### 5.3. Results for All NPCSs Combined

SENTIERI project assessed also the overall mortality profile in all the NPCSs combined. The mortality for causes of death with *a priori* Sufficient or Limited evidence of causal association with the environmental exposure showed, for the period 1995–2002, 3,508 excess deaths for all causes, corresponding to 439 deaths/year; the number of excess deaths was 1,321 for respiratory diseases, 898 for lung cancer, and 588 for pleural neoplasms. When considering excess mortality with no restriction to causes of death with *a priori* Sufficient or Limited evidence of causal association with the environmental exposure, the number of excess deaths for all causes was 9,969 (SMR 102.5, about 1,200 excess deaths/year); the excess was 4,309 for all neoplasms (about 538 excess deaths/year), 1 887 for circulatory system diseases, and 600 for respiratory system diseases.

## 6. Discussion

The ecological approach used in SENTIERI does not allow to draw firm conclusions on the causal relationships between residential exposure and health status in residents in a contaminated site; furthermore, the causal inference might be complicated for causes with multifactorial etiology in areas with multiple sources of different pollutants and concurrent presence of air pollution from urban areas. Notwithstanding these difficulties, in a number of instances, the *a priori *evaluation of the epidemiological evidence as carried out in SENTIERI reinforced the findings and strengthened the case of an etiological role to some environmental exposures. This has varying degrees of persuasiveness for example, an increased lung cancer and respiratory disease risk was observed in sites hosting refineries and petrochemical plants, suggesting the need for further studies; the ascertained exposure-disease association between pleural neoplasm mortality and asbestos was confirmed in sites with documented presence of asbestos and asbestos-like fibres [7]. Another aspect which could increase the persuasiveness of environmental-related health effects is the identification of raised health risks in children living in contaminated sites. In the 44 combined NPCSs, among children 0-1 year old, mortality from all causes and from perinatal conditions was, respectively, 4% (3,328 cases) and 5% higher (1,903 cases) than the Italian reference population, and the overall mortality was significantly increased in one or more age groups (0-1, 0–14, and 0–19 years) among children living in 11 (25%) NPCSs [13].

The value of an ecological study like SENTIERI should be measured, as recently suggested [14], against the baseline level of knowledge; in this context SENTIERI contribution can be considered high given the absence of systematic and standardized epidemiological investigations of the health impact of residents in NPCSs. 

Exposure ascertainment is a key phase in ecological environmental investigations; the exposures affecting the study population should ideally be described in detail, while in practice a number of limitations affect this crucial aspect in most studies.

In some investigations the exposure/s is a time-bound event in a limited geographical area, leading to a point source emission of a limited number of contaminants whose nature has been identified and whose toxicological properties can be partially known. Events such as the explosion of Seveso in 1976 [15] and Bhopal in 1984 belong to this category [16]. More frequently the environment has been progressively and perhaps surreptitiously contaminated by a heterogeneous mixture of pollutants originating from industry (often a variety of industrial activities) or waste treatment/disposal activities. In this case several environmental matrices are contaminated over a period of years, leading to multiple sources of exposure to a variety of exogenous agents, possibly changing qualitatively or quantitatively overtime. Often, the available exposure information is indirect and qualitative. For example, in SENTIERI project the sources of environmental exposures were abstracted from the legislative Decrees defining sites' boundaries, and chemical industry, petrochemicals and refineries plants, steel plants, power plants, mines and/or quarries, harbour areas, asbestos or other mineral fibres, landfills, and incinerators subsequently coded. Such data are insufficient to give a full picture of space and time distribution and variability of the exposures. In addition, for most NPCSs no information is available on sources of exposure that can have a health impact, such as concurrent air pollution from road traffic and exposures in the occupational setting. 

Another limitation in exposure ascertainment lies in the implicit assumption that all residents in the area under investigation experience the same exposures, while exposure variability is likely to be substantial, due to many factors (e.g., concentration of contaminants and their diffusion to soil and water, distance of residence from polluting sources). The possible consequences of such nondifferential exposure misclassification are complex, and direction of the resulting bias is not predictable [17].

An additional limitation in exposure ascertainment derives from the territorial size and the population dimension of the areas at study for which vital statistics are available. Whatever the administrative boundaries are, they hardly correspond to the distribution of environmental pollutants, so that the misclassification of exposure (and loss of statistical power) is common.

As far as outcome measures are concerned, many studies in polluted areas consider mortality, based on death records. However, the analysis of hospital discharge records, *ad hoc* registry data of specific pathologies (e.g., cancer, congenital malformations) can give a better picture of the health profile of residents in NPCSs. 

Each vital statistics is able to provide information only about the events that is designed to record, and databases need to be validated for use in epidemiological studies.

In the majority of countries death rates from all causes are unlikely to be biased because reporting the event of death is exhaustive. Therefore the overall mortality, which is an important indicator of conditions of life, can be analyzed with confidence [18]. In Italy mortality data are available for the whole country, and validity of cause of death certification has been documented for specific diseases [19–22].

In most European countries Hospital Discharge Records (HDRs) are indicators of hospital activity, and their main use is for administrative purposes [23]: validity when employed in ecologic studies has not been systematically evaluated. Some Italian reports [24, 25] comment on some critical aspects of this novel utilization of HDRs.

The analysis of cancer incidence and congenital malformations data in environmental epidemiology investigations can be considered, subject to validity evaluation. In Italy in the context of contaminated sites congenital anomalies have been used in a descriptive study [26] and the investigation of cancer incidence has been planned [27].

Mortality statistics and hospital discharge statistics have been using the succeeding versions of the International Classification of Diseases (ICD) that guarantees homogeneity and comparability of results obtained in different places. However, particularly for cancer, the resolution power of the ICD is far lower than that of classifications based on pathological diagnosis such as the succeeding versions of the International Classification of Diseases for Oncology (ICD–O). Some associations between environmental agents and cancer are limited to specific pathological variants of the disease (e.g., wood dust and adenocarcinoma of the nose and nasal sinuses); for populations served by cancer registries where the histological type of cancer is systematically recorded this problem is solved.

In environmental health studies factors as socioeconomic status, occupational exposures, and individual lifestyles can have an etiologic role on the health effects under study thus possibly confounding the exposure-disease relationships. Socioeconomic status is a determinant of health and disease. Since the mid-1990s ecologic studies of environment and health in UK adjusted for deprivation using Census data; for a review refer to Pasetto 2010 [11]. To account for deprivation in SENTIERI project mortality data were analyzed both crude and adjusted [28] based on an *ad hoc* deprivation index (SENTIERI DI). 

Occupational exposures are potential confounders in ecological studies of environment and health; individual based studies may be needed to disentangle environmental and occupational risk. 

The present paper referred as a common thread to SENTIERI project, in its main components of mortality study and *a priori* evaluation of the epidemiological evidence. The main limitations of the overall approach have been listed. Its major strengths are the standardization of the mortality analysis and NPCSs classification in terms of environmental exposure which allow the study of all NPCSs in one country; the *a priori* evidence evaluation to comment and interpret study results is a key characterizing element of the project. Additional assets are that the mortality analysis can be updated and other vital statistics data can be analysed; also the *a priori *evidence evaluation can be brought up to date following the established criteria and procedures. 

## 7. Concluding Remarks

The SENTIERI approach allows the description of the health status of populations living in the Italian NPCSs. Furthermore, it is suitable for an overall analysis of data from different NPCSs and comparative analysis of data from NPCSs with the same contamination sources.

The SENTIERI approach is, in its essence, a tool to describe the health profile of residents in NPCSs to document ascertained or suspected associations with local environmental risks; it does not require an *ad hoc* data collection [3]. The approach can also be of value for health surveillance activities in NPCSs (possibly analysing different outcomes); in addition it can contribute to etiological evaluation of cause-effect associations if additional data from biomonitoring investigations, risk assessment studies, and individual-based epidemiological studies are available.

The links between environmental exposures and health effects depend on the environmental pollutants and diseases being considered but are also influenced by factors such as genetic constitution, age, nutrition and lifestyles, occupation, and socioeconomic factors such as poverty and level of education. Identifying these relationships is therefore challenging; however, the effort is often worthwhile as it may help to redefine priorities and unlock resources [29].

Other strengths of the SENTIERI approach are that the *a priori* evidence evaluation and mortality analysis can be updated [30]; other health outcomes in addition to mortality can be analyzed, for example, cancer incidence [27], morbidity, and adverse reproductive effects; also the *a priori* evaluation can be carried out for *environmental exposures *different from the ones in SENTIERI project.

Notwithstanding the remarkably laborious activities required to set up a national project such as SENTIERI, major benefits in terms of quality and quantity of findings, and a favourable cost/gain balance can be expected inasmuch as this becomes a permanent system of epidemiological observation on health of residents in NPCSs.

## Figures and Tables

**Table 1:** Evaluation of the epidemiological evidence of the association between cause of death and *environmental exposures*.

Sufficient (S) Sufficient to infer the presence of a causal association	(i) the Sufficient evaluation relies on one or more *primary sources* or on the data they provide for such evaluation
	or
	(ii) Quantitative meta-analyses provide data for an evaluation classifiable as Sufficient

Limited (L) Limited but not sufficient to infer the presence of a causal association	(i) one or more *primary sources*/quantitative meta-analyses/reviews/multicentric studies/two or more single studies report the existence of a causal association but they do not express a Sufficient evaluation, or they do not provide data for such evaluation

Inadequate (I) Inadequate to infer the presence or the absence of a causal association	(i) some *primary sources* study the causal association but they disagree about the evaluation (conflicting evidence)
	or
	(ii) some quantitative meta-analyses/reviews/multicentric studies/two or more single studies analyze the association but they disagree about the evaluation (conflicting evidence)
	or
	(iii) some *primary sources*/quantitative meta-analyses/reviews/multicentric studies/two or more single studies analyze the causal association but none of them reports its existence
	or
	(iv) the available studies are not consistent (conflicting evidence)
	or
	(v) only one single study analyzing the causal association is available

**Table:** (a) Environmental exposures in NPCSs

Cause of death	Chemical plant*	Petrochemical plant and refinery	Steel plant	Electric power plant	Mine and/ or quarry	Harbour area	Asbestos or other mineral fibers	Landfill	Incinerator
All ages									

Malignant neoplasms of trachea, bronchus, and lung	I	L	I	L	I	I	L	I	L
Malignant neoplasms of pleura		I	I	I	S	L	S		
Diseases of the respiratory system	L	L	L	L	I	L		I	I
Asthma	L	L	L	L		L		I	I

Up to 14 years old									

Asthma	L	L	L	L				I	I

**Table:** (b) Other exposures

Cause of death	Air pollution	Active smoking	Passive smoking	Alcohol	Socioeconomic status	Occupation
All ages						

Malignant neoplasms of trachea, bronchus, and lung	S	S	S	I	S	S
Malignant neoplasms of pleura	L					S
Diseases of the respiratory system	L onset/S wors	S onset/wors	L onset/wors	S	L	S
Asthma	L onset/S wors	S onset/wors	L onset/wors	L	L	S

Up to 14 years old						

Asthma			S onset/wors		L	

**Table 3:** Mortality for the main causes of death. Number of observed cases (OBS), standardized mortality ratio crude (SMR), and adjusted for deprivation (SMR DI), 90% CI, 90% confidence interval; regional reference (1995–2002). Males and females.

NPCS: SASSUOLO-SCANDIANO						
Cause	Males			Females		
	OBS	SMR (90% CI)	SMR DI (90% CI)	OBS	SMR (90% CI)	SMR DI (90% CI)
All causes	3677	105 (102–108)	106 (103–109)	3131	102 (99–105)	102 (99–105)
All neoplasms	1241	101 (96–106)	102 (97–107)	818	93 (88–99)	95 (90–101)
Diseases of the circulatory system	1402	109 (105–114)	110 (105–115)	1416	103 (99–108)	102 (98–107)
Diseases of the respiratory system	261	118 (107–131)	120 (108–133)	160	102 (89–116)	105 (92 –120)
Diseases of the digestive system	145	106 (92–122)	107 (93–123)	149	119 (103–136)	120 (104–137)
Diseases of the genitourinary system	35	95 (70–126)	95 (71–126)	39	116 (87–151)	117 (88–152)

**Table 4:** Number of observed cases (OBS), standardized mortality ratio crude (SMR), and adjusted for deprivation (SMR DI); 90% CI, 90% confidence interval; regional reference (1995–2002). Males and females. Causes with Sufficient or Limited evidence of association with the *environmental exposures* in SASSUOLO-SCANDIANO NPCS (chemical industry).

NPCS: SASSUOLO-SCANDIANO												
Cause	Males			Females			*Environmental exposures* in the NPCS*	*Other exposures *				
	OBS	SMR (90% CI)	SMR DI (90% CI)	OBS	SMR (90% CI)	SMR DI (90% CI)		Air pollution	Active smoking	Passive smoking	Alcohol	Occupation
Malignant neoplasm of stomach	118	106 (90–124)	103 (88–121)	70	94 (76–114)	89 (72–109)	C	I	S	I	I	I
Malignant neoplasm of colon and rectum	113	91 (77–106)	92 (78–107)	79	78 (64–94)	81 (66–97)	C	**	I	I	S	I
Diseases of the respiratory system	261	118 (107–131)	120 (108–133)	160	102 (89–116)	105 (92–120)	C	L ons/S wor	S ons/wor	L ons/wor	S	S
Asthma	10	216 (117–367)	206 (112–349)	7	147 (69–275)	151 (71–284)	C	L onss/S wor	S ons/wor	L ons/wor	L	S

IPS *environmental exposures*: C: production of chemical substances, P&R: petrochemical plant and/or refinery, S: steel plant, E: electric power plant, M: mine or quarry, HA: harbour area, A: asbestos or other mineral fibres, L: landfill, I: incinerator.

Legend of the evaluation of the evidence: S: Sufficient to infer the presence of a causal association, L: Limited but not sufficient to infer the presence of a causal association I: Inadequate to infer the presence or the absence of an association, S ons/wor: sufficient onset and worsening, L ons/S wor: limited onset/sufficient worsening, L ons/wor: limited onset and worsening, *sufficient or limited evidence, **not applicable.

**Table 5:** Number of observed cases (OBS), standardized mortality ratio crude (SMR), and adjusted for deprivation (SMR DI); 90% CI, confidence interval; regional reference (1995–2002). Causes with Sufficient or Limited evidence of association with the *environmental exposures* in SASSUOLO-SCANDIANO NPCS (chemical industry).

NPCS: SASSUOLO-SCANDIANO									
Cause (age groups)				*Environmental exposures* in the NPCS*	Other exposures				
	OBS	SMR (90% CI)	SMR DI (90% CI)		Air pollution	Active smoking	Passive smoking	Alcohol	Occupation
Congenital anomalies (all ages)	25	138 (96–193)	144 (100–201)	L	l	**	L	L	I
Certain conditions originating in the perinatal period (0-1)	14	74 (44–115)	74 (45–115)	C, L	L	**	S	I	I
Asthma (0–14)	<3			C	L ons/S wor	**	S ons/wor	**	**

IPS *environmental exposures*: C: production of chemical substances, P&R: petrochemical plant and/or refinery, S: steel plant, E: electric power plant, M: mine or quarry, HA: harbour area, A: asbestos or other mineral fibres, L: landfill, I: incinerator.

Legend of the evaluation of the evidence: S: Sufficient to infer the presence of a causal association, L: Limited but not sufficient to infer the presence of a causal association I: Inadequate to infer the presence or the absence of an association, S ons/wor: sufficient onset and worsening, L ons/S wor: limited onset/sufficient worsening, L ons/wor: limited onset and worsening, *sufficient or limited evidence, **not applicable.

## Glossary

Attribute: a factor that is an intrinsic characteristic of the individual person, animal, plant, or other type of organism under study (e.g., genetic susceptibility, age, sex, breed, and weight).
Contaminated site: area hosting or having hosted human activities which have produced or might produce environmental contamination of soil, surface or ground water, air, and food chain, resulting or being able to result in human health impacts.
Cause of disease: a risk factor (e.g., characteristic, behavior, or event) that directly influences the occurrence of a disease. Reducing such a factor among a population should reduce occurrence of the disease.
Determinant: any factor that brings about change in a health condition or in other defined characteristics.
Deprivation: a state of observable and demonstrable disadvantage relative to the local community or the wider society or nation to which an individual, family, or groups belong.
Ecological study design: an approach that looks for associations between exposures and outcomes in populations rather than in individuals.
Environmental remediation: the process of removing pollution or contaminants from environmental media such as soil, groundwater, sediment, or surface water for the general protection of human health and the environment.
Exposure: having come into contact with a cause of, or possessing a characteristic that is a determinant of, a particular health problem.
Health indicator: any of a variety of measures (e.g., mortality rate) that indicate the state of health of a given population.
Incidence: a measure of the frequency with which new cases of illness, injury, or other health condition occur among a population during a specified period.
Meta-analysis: a systematic method that uses statistical techniques for combining results from different studies to obtain a quantitative estimate of the overall effect of a particular intervention or variable on a defined outcome. It produces a stronger conclusion than can be provided by any individual study.
Morbidity: the incidence of a particular disease.
Mortality rate: a measure of the frequency of occurrence of death among a defined population during a specified time interval.
Multicentric study: a study conducted at more than one research or medical centre.
Outcome: any or all of the possible results that can stem from exposure to a causal factor or from preventive or therapeutic interventions; all identified changes in health status that result from the handling of a health problem.
Population: the total number of inhabitants of a geographic area or the total number of persons in a particular group (e.g., the number of persons engaged in a certain occupation).
Risk assessment: a method to characterize the nature and magnitude of health risks to humans (e.g., residents, workers, and recreational visitors) and ecological receptors (e.g., birds, fish, and wildlife) from chemical contaminants and other stressors that may be present in the environment.
Risk factor: an aspect of personal behavior or lifestyle, an environmental exposure, or a hereditary characteristic that is associated with an increase in the occurrence of a particular disease, injury, or other health condition.
Systematic review: a critical assessment and evaluation of all research studies that address a particular clinical issue. A set of specific criteria is used in an organized method of locating, assembling, and evaluating a body of literature on a particular topic.
Standardized mortality ratio: ratio between the observed number of deaths in a study population and the number of deaths expected, based on the age- and sex-specific rates in a standard population and the age and sex distribution of the study population.
Validity: the degree to which a measurement, questionnaire, test, or study or any other data-collection tool measures what is intended to measure.
